# Clinical Predictors of Fatal Outcomes from Human Leptospirosis, Thailand, 2015–2024

**DOI:** 10.3201/eid3207.260014

**Published:** 2026-07

**Authors:** Umaporn Limothai, Nathan E. Stone, Sasipha Tachaboon, Janejira Dinhuzen, Jason W. Sahl, Theerapon Sukmark, Chayomon Dokpong, David M. Wagner, David A. Haake, Nattachai Srisawat

**Affiliations:** Chulalongkorn University Faculty of Medicine, Center of Excellence in Critical Care Nephrology, Bangkok, Thailand (U. Limothai, S. Tachaboon, J. Dinhuzen, N. Srisawat); King Chulalongkorn Memorial Hospital–Thai Red Cross Society, Excellence Center for Critical Care Nephrology, Bangkok (U. Limothai, S. Tachaboon, J. Dinhuzen, N. Srisawat); Northern Arizona University Pathogen and Microbiome Institute, Flagstaff, Arizona, USA (N.E. Stone, J.W. Sahl, D.M. Wagner); Thungsong Hospital, Nakhon Si Thammarat, Thailand (T. Sukmark); Khukhan Hospital, Sisaket, Thailand (C. Dokpong); Veterans Affairs Greater Los Angeles Healthcare System, Los Angeles, California, USA (D.A. Haake); University of California David Geffen School of Medicine, Los Angeles (D.A. Haake); University of Pittsburgh School of Medicine, Pittsburgh, Pennsylvania, USA (N. Srisawat); Royal Society of Thailand Academy of Science Bangkok (N. Srisawat)

**Keywords:** leptospirosis, bacteria, bacterial infections, mortality predictors, leptospiremia, prognostic biomarkers, Leptospira interrogans, clonal group 272, zoonoses, Thailand

## Abstract

Early predictors of fatal leptospirosis and the role of pathogen lineages remain poorly defined, limiting clinical risk stratification, genomic surveillance, and public health response in leptospirosis-endemic settings. We conducted a multicenter prospective cohort study of hospitalized patients with suspected leptospirosis in Thailand during 2015–2024. Among 459 patients with laboratory-confirmed cases, 25 (5.4%) died during hospitalization. Older age, higher total bilirubin, and higher leptospiremia were independently associated with in-hospital death, and a combined model demonstrated good discriminatory performance. We performed targeted amplicon sequencing analysis directly on clinical samples and whole-genome sequencing on available isolates. Genomic analysis identified *Leptospira interrogans* as the predominant species; clonal group 272 sequence type 34 was the predominant lineage and was observed in all patients with fatal cases for whom genomic data were available. Our findings support integration of clinical predictors and pathogen load for early risk stratification and highlight the potential value of genomic surveillance in leptospirosis-endemic settings.

Leptospirosis is a widespread zoonotic disease caused by pathogenic *Leptospira* bacteria species. It is responsible for an estimated 1.03 million cases and 58,900 deaths annually worldwide and has a mean case-fatality rate (CFR) of ≈6.8% (*1*). The disease prevalence is highest in tropical regions, where environmental exposure and occupational risk factors contribute to transmission through contaminated water, soil, and animal reservoirs (*2*–*4*). Despite its global impact, leptospirosis remains a neglected tropical disease with substantial public health and socioeconomic burdens in resource-limited settings (*5*). In Thailand, leptospirosis remains endemic, particularly among agricultural workers (*6*), and continues to cause severe illness characterized by multiorgan failure and high CFRs. National surveillance data from 2013 through 2022 reported an overall CFR of 1.46% (*7*), and our previous multicenter cohort of hospitalized patients with laboratory-confirmed cases documented a 7% mortality rate with frequent multiorgan dysfunction (*8*). Those findings highlight the substantial clinical burden of leptospirosis in endemic settings.

The wide clinical spectrum of leptospirosis probably is influenced by both host factors and the genomic diversity of *Leptospira* strains. Prior studies have shown that host clinical and inflammatory responses are major determinants of disease severity (*9*–*17*). In contrast, pathogen-related determinants, such as the contributions of specific *Leptospira* species, strains, and lineages, have been less well characterized. Infecting *Leptospira* species and serogroups or serovars have been associated with clinical syndromes (*18*), but severe disease has been reported across multiple serogroups, and our previous multicenter cohort in Thailand found no statistically significant difference in serogroup distribution between severe and nonsevere leptospirosis (*8*). That finding suggests that serogroup alone is insufficient to explain clinical phenotype and underscores the need to investigate lineage-level characteristics and host–pathogen interactions when studying severe disease. Recent molecular epidemiologic work has identified substantial genetic heterogeneity within *L. interrogans*; some lineages showed distinct geographic distributions (*18*,*19*) and potential clinical associations (*20*,*21*). However, direct evidence linking specific bacterial lineages to severe or fatal disease remains limited. A clearer understanding of pathogen diversity and how it interacts with host responses is important for improving genomic surveillance, targeted risk stratification, and public health response in endemic settings.

To address those gaps, we aimed to identify clinical, laboratory, and pathogen-load factors associated with in-hospital death in patients with confirmed leptospirosis. We also characterized *Leptospira* species and lineage diversity using targeted amplicon sequencing directly from clinical samples and whole-genome sequencing (WGS) of isolates. This integrated clinical and genomic approach is intended to improve risk prediction, inform genomic surveillance across animal reservoirs, and deepen understanding of pathogen diversity contributing to severe leptospirosis.

## Methods

### Study Design and Setting

We conducted a multicenter prospective cohort study of patients hospitalized with suspected leptospirosis during September 1, 2015–December 31, 2024, in Sisaket and Nakhon Si Thammarat, 2 leptospirosis-endemic provinces in Thailand (Appendix 1; Appendix 2). We used demographic, clinical, laboratory, and outcome data that were collected during hospitalization. The primary objectives were to identify predictors of in-hospital death and to characterize infecting *Leptospira* species and lineages among laboratory-confirmed cases. 

### Case Definition and Laboratory Confirmation

We defined laboratory-confirmed leptospirosis as >1 positive result by *lipL32* quantitative PCR (qPCR), culture, or microscopic agglutination test (MAT), as previously described (*22*). We included clinically suspected but non–laboratory-confirmed patients from the same cohort as a comparator group for clinical outcome analyses. We defined severe leptospirosis by death, intensive care unit (ICU) admission, mechanical ventilation, pulmonary hemorrhage, or organ failure on the basis of a modified Sequential Organ Failure Assessment score (*23**–**25*) (Appendix 1).

### DNA Extraction and qPCR Detection

We extracted total DNA from whole blood or urine specimens. We detected *Leptospira* DNA by using qPCR targeting the *lipL32* gene, as previously described (*26*) (Appendix 1).

### Genomic Subset and Sequencing Workflow

**Figure 1:**
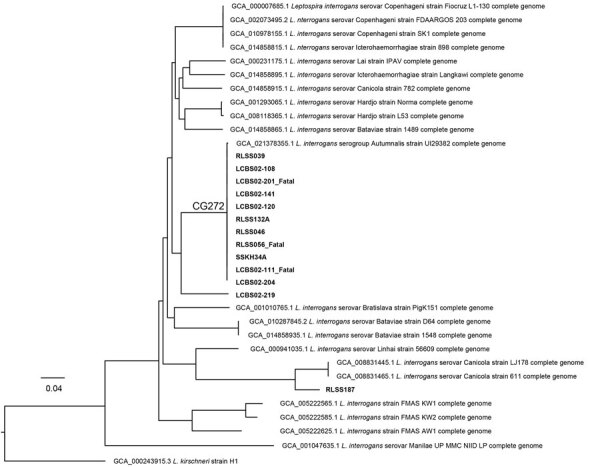
Whole-genome phylogeny of *Leptospira interrogans* isolates, Thailand, 2015–2024. Maximum-likelihood phylogenetic tree was generated by using 102,654 variable sites across a 3.9-Mb core genome of 13 *L. interrogans* isolates from this study (in bold font) plus 21 publicly available *L. interrogans* reference genomes. The tree was rooted with *L. kirschneri*. Eleven of the 13 isolates clustered within CG272, including 3 from patients with fatal cases. The remaining 2 isolates grouped outside CG272, indicating the presence of additional *L. interrogans* lineages in the region. Scale bar represents nucleotide substitutions per site. CG272, clonal group 272.

Among 473 laboratory-confirmed cases, to ensure sufficient bacterial DNA for genotyping we selected DNA extracts from 93 whole blood samples and 2 urine samples with *lipL32* qPCR cycle threshold values <35 for targeted amplicon sequencing by using the *Leptospira* AmpSeq (amplicon sequencing) assay (*27*). To minimize selection bias, we randomly selected samples meeting the cycle threshold with clinical severity blinded to the laboratory team. The selection included qPCR-positive patients across the clinical severity spectrum. In parallel, we performed WGS on 13 *L. interrogans* whole blood isolates. Seven patients had both AmpSeq and culture isolates available, enabling direct cross-validation between methods. Baseline characteristics of the genotyped subset did not meaningfully differ from the overall cohort, supporting the representativeness of the subset. We have summarized the study design and sample selection (Appendix 1 Figure 1). Phylogenetic analyses included publicly available *L. interrogans* reference genomes representing diverse lineages, together with *L. kirschneri* as an outgroup, to provide evolutionary context for lineage assignment (Appendix 1).

### Statistical Analysis

We performed group comparisons, logistic regression, receiver operating characteristic analysis, and bootstrap internal validation by using standard methods (Appendix 1). We excluded 14 laboratory-confirmed cases with missing clinical data from the primary analysis.

## Results

### Study Population and Case Confirmation

We screened a total of 641 patients with suspected leptospirosis (Appendix 1 Figure 1). Of those, 473 (73.8%) were laboratory-confirmed based on >1 positive diagnostic test: blood qPCR (385 [81.4%]), urine qPCR (195 [41.2%]), culture (22 [5.1%]), or MAT (179 [38.3%]). After excluding 14 confirmed cases because of missing clinical data, 459 patients remained, and we included them in the primary analysis. Among those, 434 (94.6%) survived and 25 (5.4%) died during hospitalization, corresponding to an overall in-hospital CFR of 5.4% among patients with laboratory-confirmed cases. 

The median age of the study population was 47 years, and most patients were male (82.9% vs. 17.1% female) (Appendix 1 Table 1). Patients who died were significantly older than survivors (median 60 years vs. 47 years; p = 0.007). The median time from fever onset to hospital admission was 3 days in both groups. Most participants were agricultural workers (70.6%), and the percentage was significantly higher among patients with fatal cases compared with survivors (88.0% vs. 69.6%; p = 0.049). Flood exposure was common (85.0% overall) and similar between groups. Active smoking also was frequent in the cohort (45.2%) but did not differ significantly between survivors and patients with fatal cases (45.4% vs. 41.7%; p = 0.720). The prevalence of other underlying conditions was low and comparable between groups. At admission, patients with fatal cases had markedly lower blood pressures compared with survivors, including lower systolic blood pressure (median 93.0 vs. 110.0 mm Hg), diastolic blood pressure (53.0 vs. 64.0 mm Hg), and mean arterial pressure (66.7 vs. 80.0 mm Hg). Most clinical symptoms were comparable between groups, although headache was less common among patients with patients with fatal cases (p = 0.046).

Laboratory findings revealed pronounced abnormalities among patients with fatal cases, including more severe thrombocytopenia (platelets 30.0 vs. 128.0 × 10^3^/µL; p<0.001), higher levels of blood urea nitrogen (51.0 vs. 17.0 mg/dL; p<0.001) and creatinine (4.6 vs. 1.2 mg/dL; p<0.001), and markedly reduced estimated glomerular filtration rate (17.6 vs. 71.6 mL/min/1.73 m^2^; p<0.001). Patients with fatal cases also exhibited more severe hepatic dysfunction, having significantly higher total bilirubin (4.1 vs. 1.1 mg/dL; p<0.001), direct bilirubin (4.2 vs. 0.5 mg/dL; p<0.001), and elevated aspartate aminotransferase levels (141.0 vs. 53.0 U/L; p<0.001) and lower serum albumin (2.6 vs. 3.4 g/dL; p<0.001) and total protein levels (6.4 vs. 6.9 g/dL; p = 0.004). We also observed lower bicarbonate levels in patients with fatal cases (17.0 vs. 23.0 mEq/L; p<0.001). Regarding diagnostics, a higher proportion of patients with fatal cases had positive blood qPCR (96.0% vs. 73.5%; p = 0.012) and significantly higher leptospiremia levels (median 4.5 vs. 2.9 log_10_ copies/mL, p<0.001).

### Clinical Outcomes and Organ Dysfunction

Among 459 hospitalized patients with confirmed leptospirosis, acute complications and organ dysfunction were common (Table 1). Nearly 1 in 5 patients required ICU admission (19.3%), and 54 patients (11.9%) required mechanical ventilation. Pulmonary hemorrhage occurred in 48 patients (10.6%).

**Table 1:** Clinical outcomes of 459 hospitalized patients with confirmed leptospirosis, Thailand, 2015–2024*

Outcome	No. (%) patients
ICU admission	87 (19.3)
Mechanical ventilation	54 (11.9)
Pulmonary hemorrhage	48 (10.6)
Cardiovascular SOFA >3†	84 (18.6)
Coagulation SOFA >3†	113 (25.1)
Renal SOFA >3†	75 (16.7)
Hepatic SOFA >3†	43 (9.8)
Multiorgan failure	105 (22.9)
Severe leptospirosis‡	197 (43.7)
In-hospital death§	25 (5.4)

*ICU, intensive care unit; SOFA, Sequential Organ Failure Assessment score. †Organ-specific SOFA subscores >3 indicate severe dysfunction. ‡Defined as death, ICU admission, mechanical ventilation, pulmonary hemorrhage, or organ failure (modified SOFA >3). §Defined as death during hospitalization for leptospirosis.

Organ-specific severe dysfunction, defined as modified Sequential Organ Failure Assessment subscores >3, also was frequent: cardiovascular dysfunction in 84 (18.6%) patients, coagulation dysfunction in 113 (25.1%), renal dysfunction in 75 (16.7%), and hepatic dysfunction in 43 (9.8%). We documented multiorgan failure in 105 (22.9%) patients. Overall, 197 (43.7%) patients met criteria for severe leptospirosis. The overall in-hospital CFR was 5.4% (25/459 patients). Clinically suspected but non–laboratory-confirmed patients from the same cohort generally had less severe outcomes, including a lower in-hospital CFR (3.0%) (Appendix 1 Table 2).

For patients with fatal cases, length of hospitalization varied widely, reflecting both early and late deaths (Appendix 2 Table 1). The most frequently documented causes of death included septic shock, pulmonary hemorrhage, acute respiratory failure, metabolic acidosis, acute kidney injury, and disseminated intravascular coagulation.

### Independent Predictors of In-Hospital Death in Leptospirosis Case-Patients

In the univariate analyses (Table 2), several admission variables were significantly associated with in-hospital death. Those variables included older age, lower mean arterial pressure, lower platelet count, higher creatinine, higher total bilirubin, lower albumin, lower bicarbonate, and higher leptospiremia (p<0.001 for all).

**Table 2:** Factors associated with in-hospital death in patients with confirmed leptospirosis, Thailand, 2015–2024

Variable	Unadjusted OR (95% CI)	p value	Adjusted OR (95% CI)	p value
Age	1.03 (1.01–1.06)	0.013	1.05 (1.01–1.09)	0.022
Sex	1.09 (0.36–3.26)	0.880		
Days of fever at admission	0.95 (0.77–1.16)	0.604		
Mean arterial pressure	0.95 (0.92–0.98)	<0.001	0.99 (0.96–1.02)	0.446
Platelet count	0.75 (0.66–0.86)	<0.001	0.99 (0.97–1.00)	0.056
Creatinine level	1.34 (1.16–1.54)	<0.001	0.97 (0.75–1.25)	0.791
Total bilirubin	1.11 (1.05–1.18)	<0.001	1.09 (1.01–1.19)	0.039
Albumin level	0.21 (0.10–0.43)	<0.001	0.57 (0.20–1.65)	0.304
Bicarbonate level	0.84 (0.77–0.91)	<0.001	0.91 (0.80–1.04)	0.158
Leptospiremia	2.12 (1.54–2.91)	<0.001	1.87 (1.26–2.75)	0.002

*Variables with p<0.10 in univariate analysis were considered for inclusion in the multivariable model. OR, odds ratio.

In the multivariable logistic regression model, age, total bilirubin, and leptospiremia remained independently associated with in-hospital death. Each 1-year increase in age was associated with a 5% increase in the odds of death (adjusted OR [aOR] 1.05 [95% CI 1.01–1.09]; p = 0.022). Higher total bilirubin on admission also was associated with increased death (aOR 1.09 [95% CI 1.01–1.19]; p = 0.039). Of note, higher leptospiremia was a strong independent predictor of death; each 1 log_10_ increase in bacterial load increased the odds of death by nearly 2-fold (aOR 1.87 [95% CI 1.26–2.75]; p = 0.002).

To further evaluate the discriminatory performance of these predictors, we performed receiver operating characteristic analysis. Age showed modest discrimination for in-hospital death (area under the curve [AUC] 0.66 [95% CI 0.56–0.77]; p = 0.003). Total bilirubin demonstrated good discrimination (AUC 0.79 [95% CI 0.70–0.88]; p<0.001), as did leptospiremia (AUC 0.78 [95% CI 0.67–0.88]; p<0.001). A combined model incorporating age, total bilirubin, and leptospiremia showed good discrimination (AUC 0.86 [95% CI 0.78–0.94]; p<0.001), outperforming each individual predictor. We summarized corresponding cutoff values and diagnostic performance metrics (Appendix 1 Tables 3). Bootstrap internal validation using 1,000 replications demonstrated stable regression coefficients and robust discrimination of the multivariable model incorporating age, total bilirubin, and leptospiremia.

### Secondary Analysis of Genomic Subset

To explore pathogen-related factors, we performed a secondary analysis in the subset of patients for whom genomic data were available. We conducted targeted amplicon sequencing on a total of 95 qPCR-positive clinical samples and conducted WGS on 13 *L. interrogans* isolates recovered from culture (Appendix 1 Figure 1). Because AmpSeq was applied directly to qPCR-positive clinical samples, we did not restrict lineage assignment to culturable isolates. We compared the characteristics of the genotyped subset with those of the overall cohort (Appendix 1 Table 4). We also summarized all metadata for all AmpSeq samples and characteristics of the sequenced isolates (Appendix 1 Table 5; Appendix 2 Tables 2). We analyzed 7 cases by using both AmpSeq and WGS, enabling direct comparison of lineage assignments. AmpSeq and WGS showed precise concordance; 6 of 7 paired samples yielded identical clonal-group classifications, and 1 sample showed evidence suggestive of mixed infection (Appendix 1 Tables 6).

In total, 84 clinical specimens yielded assignable genomic data, of which 82 (97.6%) were *L. interrogans* and 2 (2.4%) were *L. kirschneri*. Clonal-group classification identified 54 samples (64.3%) as *L. interrogans* clonal group 272 (CG272) and 30 samples (35.7%) as non-CG272. Multilocus sequence typing (MLST) and core genome MLST analyses assigned 11 of the 13 isolates to sequence type (ST) 34, corresponding to *L. interrogans* CG272, whereas 2 isolates assigned to other sequence types within *L. interrogans* (ST264 and ST76) (Appendix 1 Table 5).

**Figure 2:**
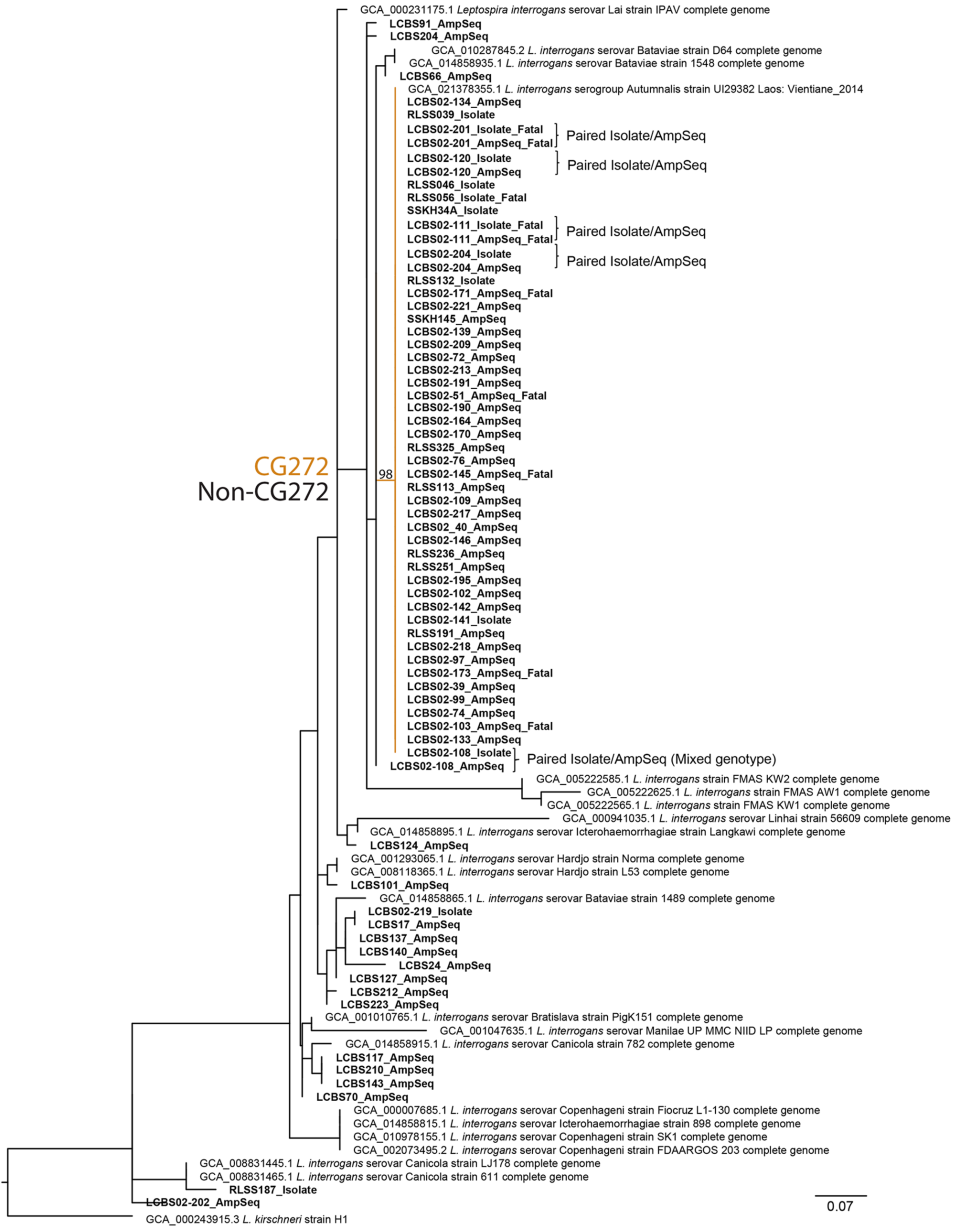
Phylogenetic analysis of *Leptospira interrogans* from AmpSeq (*27*) and isolate genome data, Thailand, 2015–2024. Maximum-likelihood phylogenetic tree was generated for high breadth of coverage AmpSeq samples (>25 loci amplified with >10× coverage) by using 59 variable sites across a 1,813-nt core genome of 57 *L. interrogans* AmpSeq samples, 13 *L. interrogans* isolates, and 21 publicly available *L. interrogans* reference genomes. The tree was rooted with *L. kirschneri*. Five isolate or AmpSeq pairs demonstrate the phylogenetic concordance between methods. A major clade corresponding to CG272 contained most of the clinical samples, including those from several patients with fatal cases, whereas the remaining samples fell into other non-CG272 lineages. A bootstrap value of 98 supports placement in the CG272 clade. CG272 and non-CG272 lineages are color-coded in gold and black. Bold font indicates sequences generated during this study. Scale bar represents nucleotide substitutions per site. AmpSeq, amplicon sequencing; CG272, clonal group 272.

Phylogenies generated from WGS data (Figure 1), high-coverage AmpSeq data (Figure 2), and the broader AmpSeq dataset (Appendix 1 Figure 2) for samples identified as *L. interrogans* yielded a consistent structure; most samples were assigned to a well-supported CG272 clade. All 9 patients with fatal cases represented in the genomic subset had isolate genomes that were located within the CG272 clade. CG272 and non-CG272 infections had broadly similar admission characteristics (Appendix 1 Tables 7). Mean leptospiremia was slightly higher among CG272 infections compared with non-CG272 infections (4.3 + SD 1.1 versus 4.0 + SD 1.1 log_10_ copies/mL; p = 0.198), although this difference was not statistically significant. We also examined the relationship between age and admission leptospiremia and found no significant correlation (Spearman ρ = 0.048; p = 0.313). 

Collectively, those results identify CG272 as the dominant *L. interrogans* lineage in the sequenced cohort and highlight that all genotyped patients with fatal cases with available genomic data were infected with isolates that clustered within that clade. Although the sample size limits formal inference, this observation warrants further investigation in larger and more systematically sampled cohorts. Of note, our exploratory analysis of single-nucleotide polymorphisms (SNPs) specific to CG272 identified 6 nonsynonymous substitutions in putative virulence-associated genes (*28*), including *Leptospira* immunoglobulin-like protein B (Appendix 2 Table 3), an immunoglobulin-like protein associated with host interaction.

## Discussion

In this multicenter cohort of 459 hospitalized patients with laboratory-confirmed leptospirosis, acute complications were frequent: 19.3% required ICU admission, 11.9% required mechanical ventilation, and 22.9% developed multiorgan failure. The in-hospital CFR was 5.4%, close to the reported global mean CFR of 6.8% (*1*), suggesting that our cohort is broadly representative of severe hospitalized leptospirosis patients rather than patient populations with unusually mild or extreme disease. For comparison, clinically suspected but non–laboratory-confirmed patients from the same hospitals had a lower in-hospital mortality rate (3.0%), indicating that confirmed leptospirosis carries a disproportionate burden of severe outcomes among patients admitted with suspected leptospirosis.

We identified 3 admission variables (age, total bilirubin, and leptospiremia) that were independently associated with in-hospital death and together provided strong discriminatory performance (AUC 0.86). Older age (*9*,*11*,*29*,*30*) and hyperbilirubinemia (*31*,*32*) are well-established predictors of severe leptospirosis and death, and our findings confirm their prognostic value in a contemporary, multicenter cohort in Thailand. Leptospiremia at admission emerged as an important pathogen-derived predictor of in-hospital death in our cohort; each 1 log_10_ increase in bacterial load nearly doubled the odds of death, and leptospiremia alone provided good discrimination (AUC 0.78). These findings suggest that combining host vulnerability (age), early hepatic dysfunction (bilirubin), and pathogen load (leptospiremia) may provide a practical and biologically plausible approach for early risk stratification in hospitalized leptospirosis patients, particularly in endemic, resource-limited settings.

The relationship between quantitative leptospiremia and disease severity has been heterogeneous across previous studies. In Sri Lanka, a 16S rRNA–gene-based qPCR did not demonstrate a clear correlation between leptospiral load and clinical manifestations or outcome (*33*). By contrast, studies from New Caledonia (*34*) and Martinique (*35*) using *lfb1*-targeted qPCR found significantly higher early leptospiremia among patients with severe disease, and a Thailand multicenter cohort employing a *lipL32* assay reported higher baseline leptospiremia was independently associated with severe leptospirosis (*8*). Our data extend this latter body of work by showing that higher admission leptospiremia, measured by using a *lipL32*-based qPCR, also is a strong independent predictor of in-hospital death. Methodologic differences in qPCR platforms (including distinct gene targets, primer–probe sets, instruments, and sample types), along with variation in infecting *Leptospira* genotypes and timing of sampling, may partly explain the inconsistent associations reported across studies. Taken together, those data support early leptospiremia as a useful, although not universally consistent, marker of severity and highlight the importance of evaluating pathogen load in parallel with other factors when investigating determinants of severe and fatal leptospirosis. This integrated approach may help clarify whether observed differences in clinical outcomes reflect variation in pathogen load, genotype, or host–pathogen interactions.

Genomic characterization revealed limited species diversity; we identified *L. interrogans* in nearly all genotyped samples, consistent with prior reports from Southeast Asia and other endemic settings (*19*,*21*,*36*,*37*). Within *L. interrogans*, CG272 (ST34) was the predominant lineage and formed a well-supported clade in both amplicon and WGS analyses. We did not observe significant differences in early clinical severity or leptospiremia between CG272 and non-CG272 infections, but all patients with fatal cases in the genomic subset had infecting isolate genomes that clustered within CG272. The genomic subset in our study was small, so this observation should be interpreted cautiously, but lineage assignment was not based solely on cultured isolates, given that targeted amplicon sequencing also was applied directly to qPCR-positive and culture-negative clinical samples. This lineage has been linked to rodent reservoirs, including both *Bandicota indica* rats and *Mus cookii* mice in northern Thailand, indicating that the lineage is not restricted to a single host species (*20*,*39*). In addition, historical and regional data further support the persistence of ST34 or CG272 across Thailand and Laos over more than a decade, including in outbreak settings and fatal infections (*19*,*21*). Together, those findings suggest that pathogen lineage may contribute to clinical heterogeneity, although whether lineage-specific factors influence disease severity remains uncertain, so the findings should therefore be considered hypothesis-generating rather than causal.

To further explore potential CG272-specific features, exploratory analysis of lineage-defining SNPs identified 6 nonsynonymous substitutions in putative virulence-associated genes, including adhesins such as *Leptospira* immunoglobulin-like protein B and *lsa30* and related host-interaction proteins (Appendix 2 Table 3). Those 6 CG272-specific variants were conserved across CG272 isolates but absent in non-CG272 strains. The functional importance of those mutations remains unknown, and no direct link to increased virulence can be inferred; however, they provide preliminary hypotheses for future mechanistic investigation.

The strengths of this study include its multicenter prospective design across 2 endemic provinces, standardized clinical assessment and management according to national guidelines, and rigorous case confirmation using qPCR, culture, and MAT. Comprehensive clinical phenotyping that included organ dysfunction scores, complications, and objective outcomes provided a robust foundation for evaluating disease severity. Of note, this study examined both host and pathogen determinants of outcome. Host factors such as age and total bilirubin were evaluated alongside pathogen factors including leptospiremia and lineage-level genomic characterization. The integration of targeted amplicon sequencing and WGS added a valuable genomic dimension that enabled detailed investigation of circulating leptospiral populations. High-resolution genomic data of this kind rarely are available in leptospirosis cohorts and enabled identification of CG272 as the predominant lineage in the sequenced subset and the only lineage observed among patients with fatal cases for whom genomic data were available. Further, the development of an admission death prediction model with excellent discriminatory performance (AUC 0.86) highlights the potential for early and practical risk stratification in routine clinical care.

One limitation of this study is that because amplicon-based sequencing and culture isolation require a minimum bacterial load, we restricted genomic analyses to samples with sufficient pathogen quantity or to cases with available isolates. That approach might lead to underrepresentation of certain lineages or low pathogen-load infections, and therefore the associations between lineage and clinical outcomes should be interpreted with caution. Larger and more systematically sampled genomic datasets, including expanded WGS of non-CG272 lineages, will be needed to clarify whether the observed clustering of isolates from patients with fatal cases within CG272 persists after minimizing potential technical and sampling biases. To reduce selection bias, we randomly selected samples with severity status blinded. In addition, we did not design the exploratory analysis of CG272-specific SNPs to establish functional relevance, and the observed variants require further functional characterization. In addition, we did not observe significant differences in leptospiremia or early clinical severity markers between CG272 and non-CG272 infections. However, those comparisons were limited by the small number of sequenced samples and the limited number of deaths, which reduced statistical power to detect modest lineage-associated effects. Larger genomic datasets will be needed to clarify whether CG272 is associated with differences in bacterial load, host response, or clinical outcomes. Moreover, because this study was conducted in district-level hospitals, the findings may not be fully generalizable to community cases, milder infections, or healthcare settings with different referral pathways. Further, although this was a prospective cohort study, there may be relevant factors that were not measured, such as additional host characteristics, environmental exposures, or differences in treatment, that could have influenced the results.

Our findings provide direction for integrated public health surveillance integrating human clinical, environmental, and reservoir data. Future studies should combine clinical predictors with pathogen genotype in broader populations and across different healthcare settings. Expanded genomic surveillance will be important for tracking the distribution and evolution of potentially high-risk lineages such as CG272. Integrative studies that combine host biomarkers, pathogen load, and genomic data may help clarify mechanisms of severe disease. Functional experiments also are needed to determine how specific lineages influence pathogenicity and immune responses. Further, developing practical clinical tools that incorporate both host and pathogen information may improve early recognition and management of patients in endemic regions who are at high risk.

In conclusion, this multicenter prospective study identified key host and pathogen factors associated with death in hospitalized patients with leptospirosis. Older age, elevated total bilirubin, and higher leptospiremia at admission were independently associated with in-hospital death, and a combined model demonstrated excellent discrimination. Genomic analysis identified CG272 as the predominant lineage in the sequenced subset and the only lineage observed among patients with fatal cases for whom genomic data were available, although this association remains exploratory. Our findings support the integration of clinical and pathogen data to improve early risk stratification and guide public health surveillance in endemic settings.

Appendix 1 Additional information for clinical predictors of fatal outcomes from human leptospirosis, Thailand, 2015–2024.

Appendix 2 Additional tables for clinical predictors of fatal outcomes from human leptospirosis, Thailand, 2015–2024.
