# Supplementary material for: Clinical Predictors of Fatal Outcomes from Human Leptospirosis, Thailand, 2015–2024
Source: Emerg Infect Dis. 2026 Jul;32(7):1094–103. doi: 10.3201/eid3207.260014 (PMC13322444; doi:10.3201/eid3207.260014)
Supplement: Appendix 1 — Additional information for clinical predictors of fatal outcomes from human leptospirosis, Thailand, 2015–2024. [file 26-0014-Techapp-s1.pdf]

Article DOI: https://doi.org/10.3201/eid3207.260014

EID cannot ensure accessibility for supplementary materials supplied by authors. Readers who have difficulty accessing supplementary content should contact the authors for assistance.

Clinical Predictors of Fatal Outcomes from Human Leptospirosis, Thailand, 2015–2024

Appendix 1

Supplementary Methods

Study Design and Setting

We conducted a multicenter prospective cohort study of patients hospitalized with suspected leptospirosis between September 1, 2015, and December 31, 2024, in two endemic provinces of Thailand: Sisaket (northeastern region) and Nakhon Si Thammarat (southern region). Demographic, clinical, and laboratory data were collected at enrollment and throughout hospitalization. Clinical outcomes, including complications, organ dysfunction, and in-hospital mortality, were obtained from detailed medical records. All study sites were district-level hospitals under the Thai Ministry of Public Health. These hospitals followed the same national clinical guidelines for leptospirosis management (*1*), including early empiric antibiotic therapy, standardized protocols for fluid resuscitation, and access to renal replacement therapy. Dialysis availability and referral criteria were similar across sites. The primary objectives were to identify predictors of in-hospital mortality and to characterize infecting *Leptospira* species and lineage among laboratory-confirmed cases.

The study was approved by the Central Research Ethics Committee of Thailand (CREC036/58BRM for the discovery cohort; CREC068/67BR-MED44 for the validation cohort). Written informed consent was obtained from all participants aged ≥18 years and from the parents or legal guardians of participants aged <18 years. The study adhered to the principles of the Declaration of Helsinki and Good Clinical Practice guidelines.

Case Definition and Laboratory Confirmation

Patients were classified as having laboratory-confirmed leptospirosis if they tested positive by at least one of the following criteria: quantitative PCR (qPCR) targeting *lipL32* in

blood or urine; culture isolation of *Leptospira* species from blood; or a microscopic agglutination test (MAT) demonstrating either a ≥4-fold rise in antibody titer between paired sera or a single titer of ≥1:400 (*2*). Clinically suspected but laboratory-unconfirmed patients from the same prospective cohort served as a clinically relevant control group for comparison of clinical outcomes. Laboratory-unconfirmed patients were defined as those with suspected leptospirosis but negative results on all available laboratory-confirmatory tests. Severe leptospirosis was defined as the presence of any of the following: in-hospital death, admission to the intensive-care unit (ICU), requirement for mechanical ventilation, pulmonary hemorrhage, or evidence of organ failure as indicated by a modified Sequential Organ Failure Assessment (mSOFA) score greater than 2 in any organ system (*3–5*).

DNA Extraction and Quantitative PCR Detection

Total DNA was extracted from 200 µL of whole blood or urine pellet using the High Pure PCR Template Preparation Kit (Roche Diagnostics, Germany) according to the manufacturer's instructions. Detection of *Leptospira* DNA was performed by quantitative PCR targeting the *lipL32* gene, following the primers and amplification conditions described by Stoddard et al. (*6*), with minor modifications as described in our previous publication (*7*).

Sample Selection for Genomic Analysis

Among 473 laboratory-confirmed cases, to ensure adequate bacterial DNA yield DNA extracts from 95 samples with *lipL32* qPCR cycle threshold (Ct) values <35 were selected for AmpSeq. The selection aimed to include qPCR-positive patients with a range of clinical severity. In parallel, 13 *L. interrogans* cultured isolates were subjected to whole-genome sequencing. Seven cases with available isolates were included in both analyses for cross-validation. Baseline characteristics of the genotyped subset were generally comparable to the overall confirmed cohort, indicating representativeness of the sequenced cases (Appendix 1 Table 4). The study design and sample selection are summarized in Appendix 1 Figure 1.

Targeted Amplicon Sequencing

Amplicon sequencing was performed using the *Leptospira* AmpSeq system as previously described (*8*). Briefly, 42 primer sets targeting lineage-informative and species-discriminatory loci were amplified in four multiplex PCR reactions for each sample. Amplicons were pooled for each sample and dual indexed libraries were prepared and pooled as previously described (*9*,*10*)

and sequenced on an Illumina NextSeq 1000 instrument using a 600 cycle (2x300) P1 kit (Illumina, USA). Non-template and PCR positive controls were also included. Sequencing reads were processed using the Amplicon Sequencing Analysis Pipeline (ASAP; https://github.com/TGenNorth/ASAP), which maps paired-end reads to a curated *Leptospira* reference database and counts reads assigned to each locus. Samples that amplified at ≥3 loci and with >10x coverage were assigned a species ID, except for samples LCBS02–204, LCBS02–11, LCBS69, and LCBS106, where mixed and low coverage AmpSeq reads made species identification unclear (Appendix 2 Table 2).

Whole-Genome Sequencing

Genomic DNA from 13 cultured *Leptospira* isolates was quantified using a Qubit fluorometer and assessed for integrity by Bioanalyzer. One isolate (LCBS_02_204B) with high-quality, high-molecular-weight DNA (>15 kb) underwent long-read sequencing on the PacBio Sequel II/IIe platform, whereas the remaining 12 isolates, which showed lower DNA yield or partial degradation, were processed for short-read sequencing on Illumina HiSeq/NovaSeq or MGI2000 platforms. All sequencing was performed by Novogene Co., Ltd (Singapore). SMRTbell libraries for PacBio and indexed libraries for Illumina/MGI were prepared following the manufacturers' standard protocols. Short-read libraries were generated from ~100 ng DNA fragmented to ~350 bp insert size, PCR-amplified, purified, and validated before 2 × 150 bp paired-end sequencing. Base calling and image analysis used instrument-specific software provided by each platform.

Bioinformatic Methods

Genome Assemblies

Illumina whole genome sequencing reads were assembled using meta-SPAdes v3.13.0 (*11*) with default settings. PacBio reads for sample LCBS02–204 were assembled with Flye v2.9.5 (*12*).

cgMLST clonal group (CG) and sequence type (ST) determination

Assembled genomes were queried against the cgMLST database https://bigsdb.pasteur.fr/cgi-bin/bigsdb/bigsdb.pl?db = pubmlst_*Leptospira*_seqdef&page = sequenceQuery and CGs were determined using previously established criteria [no more than 40 allelic mismatches out of 545

loci (*13*)]. The corresponding MLST (*14*) sequence type was also determined by querying against the MSLT (scheme 1) database.

Phylogenetic analyses

Single nucleotide polymorphisms (SNPs) were identified among the 13 *L. interrogans* genomes generated from cultured isolates (Appendix 1 Table 5) and 21 publicly available *L. interrogans* complete genomes (GenBank accession numbers provided in figures) by aligning reads against reference genome *L. interrogans* serovar Copenhageni strain Fiocruz L1–130 using minimap2 v2.22 (*15*) and calling SNPs from the BAM file with GATK v4.2.2 (*16*) using a depth of coverage ≥10x and a read proportion of 0.9. SNPs that fell within duplicated regions, based on a reference self-alignment with NUCmer v3.1 (*17*), were filtered from downstream analyses. All methods were wrapped by NASP v1.2.1 (*18*). The annotation of SNP mutations was performed with snpEff v5.0e (*19*). A maximum likelihood phylogeny was inferred on the concatenated SNP alignment using IQ-TREE v2.2.0.3 with default parameters (*20*), the "-fast" option, and the integrated ModelFinder method (*21*); the phylogeny was rooted with the reference genome *L. kirschneri* strain H1.

To facilitate genotyping within species for AmpSeq samples with high breadth of coverage (i.e., ≥25 loci amplified with >10x coverage) (Appendix 2 Table 2), SNPs were identified from shared regions among the AmpSeq amplicons, and the same 13 cultured isolates and 21 publicly available *L. interrogans* genomes described above. For AmpSeq reads, Illumina adaptors and universal tail sequences were first removed in ASAP and locus specific primers were trimmed using cutPrimers (https://github.com/aakechin/cutPrimers) (*22*), and then AmpSeq reads, isolate genome reads, and genome assemblies were aligned against reference genome *L. interrogans* serovar Copenhageni strain Fiocruz L1–130 using NASP v1.2.1 (*18*) and a maximum likelihood phylogeny was inferred with IQ-TREE v2.2.0.3 (*20*) as described above, except with 1000 bootstraps replicates. Five of seven isolate/AmpSeq pairs were included in this analysis to assess phylogenetic concordance between methods. To investigate a potential *L. interrogans* mixture in sample LCBS2/108, AmpSeq reads were mapped against the LCBS2/108 isolate genome assembly in NASP v1.2.1 (*18*). The read pileup BAM file was then manually inspected using Tablet (*23*) to identify mixed genotypes and identical sequence matches to the CG272 isolate genome for all amplified AmpSeq loci.

Membership to cgMLST CG272 was inferred for low coverage samples (<25 AmpSeq loci amplified with >10x coverage) by aligning *Leptospira* reads from all 76 AmpSeq samples assigned to *L. interrogans* (Appendix 2 Table 2) to CG272 representative genome *L. interrogans* serogroup Autumnalis strain UI29382 (GCA_021378355.1). SNPs were identified using NASP v1.2.1 (*18*) and a maximum likelihood phylogeny was inferred with IQ-TREE v2.2.0.3 (*20*) using the "missing data" SNP matrix output from NASP that replaces missing core genome SNPs with "N's." The phylogeny was rooted with *L. interrogans* serovar Bataviae strain 1489 (GCA_014858865.1).

Statistical Analysis

Demographic, clinical, and laboratory variables were compared between survivors and fatal cases using the chi-square or Fisher exact tests for categorical variables and the Mann–Whitney U test for continuous variables. Univariate logistic regression was used to identify admission factors associated with in-hospital mortality. Variables with $p < 0.10$ or deemed clinically relevant were considered for multivariable analysis. Because only 25 deaths occurred, multivariable logistic regression was limited to prevent overfitting, guided by the events-per-variable principle (*24*). Candidate predictors were therefore assessed based on (i) strength of univariate association, (ii) biologic and clinical plausibility, and (iii) acceptable collinearity metrics (tolerance >0.7 and variance inflation factor <2). Final model selection emphasized parsimony and model stability. Results are reported as odds ratios (ORs) with 95% confidence intervals (CIs). Candidate predictors were selected based on univariate strength, clinical plausibility, and acceptable collinearity (tolerance >0.7; VIF <2). Adjusted results are reported as odds ratios (ORs) with 95% confidence intervals (CIs). Discriminatory performance was evaluated using receiver operating characteristic (ROC) curves for individual predictors and the combined model. Bootstrap regression was used to assess the stability of regression coefficients, and Bias-corrected and accelerated (BCa) 95% CI were calculated. Model discrimination was evaluated using the AUC with bootstrap-based confidence intervals for the combined model including age, total bilirubin, and leptospiremia. All analyses were performed using SPSS Statistics 26.0 (IBM Corp., USA), and figures were generated with GraphPad Prism 9.5. A two-sided $p < 0.05$ was considered statistically significant. Fourteen laboratory-confirmed cases were excluded from the primary analysis because of missing clinical data. For the remaining cohort, descriptive and inferential analyses were conducted using available data for each variable.

References

1. Department of Disease Control, Ministry of Public Health. Guidelines for leptospirosis diagnosis and treatment. 2022 [cited 2026]. https://cimjournal.com/wp-content/uploads/2022/10/Guidelines-Leptospirosis.pdf

2. World Health Organization. Human leptospirosis: guidance for diagnosis, surveillance and control. 2003 [cited 2026 Jan 5]. https://www.who.int/publications/i/item/human-leptospirosis-guidance-for-diagnosis-surveillance-and-control

3. Moreno R, Rhodes A, Piquilloud L, Hernandez G, Takala J, Gershengorn HB, et al. The Sequential Organ Failure Assessment (SOFA) score: has the time come for an update? Crit Care. 2023;27:15. PubMed https://doi.org/10.1186/s13054-022-04290-9

4. Singer M, Deutschman CS, Seymour CW, Shankar-Hari M, Annane D, Bauer M, et al. The Third International Consensus Definitions for Sepsis and Septic Shock (Sepsis-3). JAMA. 2016;315:801–10. PubMed https://doi.org/10.1001/jama.2016.0287

5. Vincent JL, Moreno R, Takala J, Willatts S, De Mendonça A, Bruining H, et al. The SOFA (Sepsis-Related Organ Failure Assessment) score to describe organ dysfunction/failure. Intensive Care Med. 1996;22:707–10. PubMed https://doi.org/10.1007/BF01709751

6. Stoddard RA, Gee JE, Wilkins PP, McCaustland K, Hoffmaster AR. Detection of pathogenic *Leptospira* spp. through TaqMan polymerase chain reaction targeting the LipL32 gene. Diagn Microbiol Infect Dis. 2009;64:247–55. PubMed https://doi.org/10.1016/j.diagmicrobio.2009.03.014

7. Limothai U, Lumlertgul N, Sirivongrangson P, Kulvichit W, Tachaboon S, Dinhuzen J, et al. The role of leptospiremia and specific immune response in severe leptospirosis. Sci Rep. 2021;11:14630. PubMed https://doi.org/10.1038/s41598-021-94073-z

8. Stone NE, Hamond C, Clegg JR, McDonough RF, Bourgeois RM, Ballard R, et al. Host population dynamics influence *Leptospira* spp. transmission patterns among *Rattus norvegicus* in Boston, Massachusetts, US. PLoS Negl Trop Dis. 2025;19:e0012966. PubMed https://doi.org/10.1371/journal.pntd.0012966

9. Colman RE, Schupp JM, Hicks ND, Smith DE, Buchhagen JL, Valafar F, et al. Detection of low-level mixed-population drug resistance in *Mycobacterium tuberculosis* using high fidelity amplicon sequencing. PLoS One. 2015;10:e0126626. PubMed https://doi.org/10.1371/journal.pone.0126626

10. Stone NE, Hall CM, Ortiz M, Hutton SM, Santana-Propper E, Celona KR, et al. Diverse lineages of pathogenic *Leptospira* species are widespread in the environment in Puerto Rico, USA. PLoS Negl Trop Dis. 2022;16:e0009959. PubMed https://doi.org/10.1371/journal.pntd.0009959

11. Nurk S, Meleshko D, Korobeynikov A, Pevzner PA. metaSPAdes: a new versatile metagenomic assembler. Genome Res. 2017;27:824–34. PubMed https://doi.org/10.1101/gr.213959.116

12. Kolmogorov M, Yuan J, Lin Y, Pevzner PA. Assembly of long, error-prone reads using repeat graphs. Nat Biotechnol. 2019;37:540–6. PubMed https://doi.org/10.1038/s41587-019-0072-8

13. Guglielmini J, Bourhy P, Schiettekatte O, Zinini F, Brisse S, Picardeau M. Genus-wide *Leptospira* core genome multilocus sequence typing for strain taxonomy and global surveillance. PLoS Negl Trop Dis. 2019;13:e0007374. PubMed https://doi.org/10.1371/journal.pntd.0007374

14. Boonsilp S, Thaipadungpanit J, Amornchai P, Wuthiekanun V, Bailey MS, Holden MT, et al. A single multilocus sequence typing (MLST) scheme for seven pathogenic *Leptospira* species. PLoS Negl Trop Dis. 2013;7:e1954. PubMed https://doi.org/10.1371/journal.pntd.0001954

15. Li H. Minimap2: pairwise alignment for nucleotide sequences. Bioinformatics. 2018;34:3094–100. PubMed https://doi.org/10.1093/bioinformatics/bty191

16. McKenna A, Hanna M, Banks E, Sivachenko A, Cibulskis K, Kernytsky A, et al. The Genome Analysis Toolkit: a MapReduce framework for analyzing next-generation DNA sequencing data. Genome Res. 2010;20:1297–303. PubMed https://doi.org/10.1101/gr.107524.110

17. Delcher AL, Salzberg SL, Phillippy AM. Using MUMmer to identify similar regions in large sequence sets. Curr Protoc Bioinformatics. 2003;10.3. **PMID 18428693**

18. Sahl JW, Lemmer D, Travis J, Schupp JM, Gillece JD, Aziz M, et al. NASP: an accurate, rapid method for the identification of SNPs in WGS datasets that supports flexible input and output formats. Microb Genom. 2016;2:e000074. PubMed https://doi.org/10.1099/mgen.0.000074

19. Cingolani P, Platts A, Wang L, Coon M, Nguyen T, Wang L, et al. A program for annotating and predicting the effects of single nucleotide polymorphisms, SnpEff: SNPs in the genome of *Drosophila melanogaster* strain w1118; iso-2; iso-3. Fly (Austin). 2012;6:80–92. PubMed https://doi.org/10.4161/fly.19695

20. Nguyen LT, Schmidt HA, von Haeseler A, Minh BQ. IQ-TREE: a fast and effective stochastic algorithm for estimating maximum-likelihood phylogenies. Mol Biol Evol. 2015;32:268–74. PubMed https://doi.org/10.1093/molbev/msu300

21. Kalyaanamoorthy S, Minh BQ, Wong TKF, von Haeseler A, Jermiin LS. ModelFinder: fast model selection for accurate phylogenetic estimates. Nat Methods. 2017;14:587–9. PubMed https://doi.org/10.1038/nmeth.4285

22. Kechin A, Boyarskikh U, Kel A, Filipenko M. cutPrimers: A new tool for accurate cutting of primers from reads of targeted next generation sequencing. J Comput Biol. 2017;24:1138–43. PubMed https://doi.org/10.1089/cmb.2017.0096

23. Milne I, Stephen G, Bayer M, Cock PJ, Pritchard L, Cardle L, et al. Using Tablet for visual exploration of second-generation sequencing data. Brief Bioinform. 2013;14:193–202. PubMed https://doi.org/10.1093/bib/bbs012

24. Austin PC, Steyerberg EW. Events per variable (EPV) and the relative performance of different strategies for estimating the out-of-sample validity of logistic regression models. Stat Methods Med Res. 2017;26:796–808. PubMed https://doi.org/10.1177/0962280214558972

Appendix 1 Table 1. Baseline Characteristics of Patients With Confirmed Leptospirosis, Comparing Survivors and Fatal Cases

Characteristics	All (N = 459)	Survived (N = 434)	Fatal (N = 25)	Case-fatality, n/N (%)
Demographics & Timing				
Age (years)	47.0 (35.0, 59.0)	47.0 (35.0, 58.3)	60.0 (44.0, 66.5)	
BMI (kg/m^2)	21.6 (19.5, 24.2)	21.6 (19.5, 24.2)	21.8 (19.2, 24.3)	
Male gender	378 (82.9)	357 (82.8)	21 (84.0)	21/378 (5.6)
Days from fever onset to admission (days)	3.0 (2.0, 4.0)	3.0 (2.0, 4.0)	3.0 (2.0, 4.0)	
Occupational exposure				
Agriculture / field work	324 (70.6)	302 (69.6)	22 (88.0)	22/324 (6.8)
Animal exposure / livestock	2 (0.4)	2 (0.5)	0 (0.0)	0/2 (0.0)
Fishing / aquaculture	15 (3.3)	14 (3.2)	1 (4.0)	1/15 (6.7)
Flood exposure	380 (85.0)	358 (84.6)	22 (91.7)	22/380 (5.8)
Animal exposure	79 (17.7)	73 (17.3)	6 (25.0)	6/79 (7.6)
Comorbidities				
Diabetes mellitus	18 (4.0)	18 (4.2)	0 (0.0)	0/18 (0.0)
Hypertension	34 (7.5)	32 (7.5)	2 (8.0)	2/34 (5.9)
Chronic liver disease	3 (0.7)	2 (0.5)	1 (4.0)	1/3 (33.3)
Alcohol use	19 (4.2)	17 (4.0)	2 (8.0)	2/19 (10.5)
Active smoking	203 (45.2)	193 (45.4)	10 (41.7)	10/203 (4.9)
Vital signs				
Temperature (°C)	38.1 (37.0, 39.0)	38.2 (37.0, 39.0)	37.6 (36.8, 38.3)	
Systolic BP (mmHg)	110.0 (96.5, 122.5)	110.0 (99.0, 125.0)	93.0 (78.0, 107.0)	
Diastolic BP (mmHg)	63.0 (58.5, 74.0)	64.0 (59.3, 74.0)	53.0 (49.5, 68.5)	
MAP (mmHg)	79.5 (70.7, 90.0)	80.0 (71.7, 90.0)	66.7 (58.0, 84.5)	
Symptoms & Signs				
Pale	39 (8.7)	36 (8.5)	3 (12.0)	3/39 (7.7)
Stiff neck	12 (2.7)	12 (2.8)	0 (0.0)	0/12 (0.0)
Splenomegaly	3 (0.7)	3 (0.7)	0 (0.0)	0/3 (0.0)
Pulmonary crackles	16 (3.6)	15 (3.5)	1 (4.0)	1/16 (6.3)
Icteric sclera	53 (11.8)	47 (11.1)	6 (24.0)	6/53 (11.3)
Hepatomegaly	3 (0.7)	3 (0.7)	0 (0.0)	0/3 (0.0)
Pulmonary edema	6 (1.3)	6 (1.4)	0 (0.0)	0/6 (0.0)
Jaundice	50 (11.0)	46 (10.7)	4 (16.0)	4/50 (8.0)
Nausea	103 (22.7)	100 (23.3)	3 (12.0)	3/103 (2.9)
Vomiting	105 (23.1)	101 (23.5)	4 (16.0)	4/105 (3.8)
Headache	317 (69.8)	304 (70.9)	13 (52.0)	13/317 (4.1)

Characteristics	All (N = 459)	Survived (N = 434)	Fatal (N = 25)	Case-fatality, n/N (%)
Photophobia	4 (0.9)	4 (0.9)	0 (0.0)	0/4 (0.0)
Malaise / Fatigue	277 (61.1)	263 (61.4)	14 (56.0)	14/277 (5.1)
Alteration of conscious	15 (3.3)	13 (3.0)	2 (8.0)	2/15 (13.3)
Skin lesion	69 (15.2)	65 (15.2)	4 (16.0)	4/69 (5.8)
Abdominal pain	50 (11.0)	48 (11.2)	2 (8.0)	2/50 (4.0)
Gross hematuria	8 (1.8)	8 (1.9)	0 (0.0)	0/8 (0.0)
Dysuria	55 (12.1)	52 (12.1)	3 (12.0)	3/55 (5.5)
Backpain	182 (40.1)	174 (40.6)	8 (32.0)	8/182 (4.4)
Convulsion	1 (0.2)	1 (0.2)	0 (0.0)	0/1 (0.0)
Dyspnea	64 (14.1)	60 (14.0)	4 (16.0)	4/64 (6.3)
Cough	116 (25.6)	111 (25.9)	5 (20.0)	5/116 (4.3)
Hemoptysis	10 (2.2)	9 (2.1)	1 (4.0)	1/10 (10.0)
Oliguria	42 (26.9)	39 (26.0)	3 (50.0)	3/42 (7.1)
Laboratory Findings				
Hematologic parameters				
Hemoglobin (g/dL)	12.1 (10.7, 13.3)	12.1 (10.8, 13.4)	11.6 (9.3, 13.0)	
Hematocrit (%)	36.7 (33.0, 40.3)	36.7 (33.2, 40.6)	35.0 (27.8, 39.4)	
Leukocyte Count ($\times 10^3/\mu L$)	10.0 (7.3, 12.9)	10.0 (7.3, 12.9)	10.8 (6.5, 16.3)	
Neutrophils (%)	84.0 (73.0, 89.0)	84.0 (73.0, 89.0)	86.7 (75.0, 91.2)	
Lymphocytes (%)	9.1 (6.0, 16.4)	9.4 (6.0, 16.5)	6.0 (3.5, 14.5)	
Platelet count ($\times 10^3/\mu L$)	120.0 (60.0, 194.5)	128.0 (64.0, 198.0)	30.0 (23.0, 58.5)	
Renal function				
BUN (mg/dL)	18.6 (13.0, 37.0)	17.0 (12.4, 33.3)	51.0 (29.5, 81.9)	
Creatinine (mg/dL)	1.3 (0.9, 2.2)	1.2 (0.9, 2.0)	4.6 (1.8, 5.8)	
eGFR (mL/min/1.73 m^2)	68.7 (34.6, 94.0)	71.6 (38.4, 95.1)	17.6 (9.8, 46.3)	
Hepatic function				
Total bilirubin (mg/dL)	1.2 (0.7, 2.6)	1.1 (0.7, 2.4)	4.1 (2.1, 11.5)	
Direct bilirubin (mg/dL)	0.6 (0.3, 1.7)	0.5 (0.3, 1.6)	4.2 (1.3, 9.4)	
AST (U/L)	57.5 (35.0, 117.3)	53.0 (34.0, 110.5)	141.0 (103.0, 217.0)	
ALT (U/L)	51.0 (29.0, 86.8)	49.0 (29.0, 86.0)	64.0 (44.0, 134.0)	
Albumin (g/dL)	3.4 (2.8, 3.8)	3.4 (2.9, 3.8)	2.6 (2.2, 3.2)	
Total protein (g/dL)	6.8 (6.1, 7.5)	6.9 (6.1, 7.5)	6.4 (5.0, 6.9)	
Electrolytes				
Sodium (mEq/L)	134.0 (131.0, 137.0)	134.0 (131.0, 137.0)	134.0 (131.0, 137.5)	
Potassium (mEq/L)	3.6 (3.3, 3.9)	3.6 (3.3, 3.9)	3.7 (3.3, 4.2)	
Chloride (mEq/L)	98.0 (94.0, 101.0)	98.0 (94.7, 101.0)	97.0 (92.5, 100.0)	
Bicarbonate (mEq/L)	23.0 (19.9, 25.5)	23.0 (20.0, 26.0)	17.0 (14.4, 23.5)	
Diagnostics				
Blood qPCR (LipL32)	343 (74.7)	319 (73.5)	24 (96.0)	24/343 (7.0)
Leptospiremia (log_{10} genome copies/mL)	3.0 (1.0, 3.8)	2.9 (1.0, 3.6)	4.5 (3.5, 5.4)	
Urine qPCR (LipL32)	164 (38.8)	155 (38.0)	9 (60.0)	9/164 (5.5)
Blood culture	22 (5.3)	19 (4.8)	3 (15.8)	3/22 (13.6)

Data are presented as median (IQR) for continuous variables and n (%) for categorical variables. P values were calculated using the Mann–Whitney U test for continuous variables and χ^2 or Fisher exact test for categorical variables. Bold values indicate $p < 0.05$. Case-fatality was calculated as the proportion of in-hospital deaths within each subgroup. This table is descriptive; univariable and multivariable associations with in-hospital mortality are presented in Table 3. Percentages for some variables were calculated based on available data.

Appendix 1 Table 2. Comparison of baseline clinical and laboratory characteristics between laboratory-confirmed leptospirosis cases and clinically suspected but laboratory-unconfirmed controls

Variable	Laboratory-confirmed leptospirosis (n = 459)	Laboratory-unconfirmed controls (n = 168)
Demographic characteristics		
Age, years	47.0 (35.0, 59.0)	49.0 (38.0, 60.0)
Male sex, n (%)	378 (82.9)	118 (70.2)
Duration of illness before admission, days	3.0 (2.0, 4.0)	3.0 (2.0, 4.0)
Hematologic parameters		
Hemoglobin (g/dL)	12.1 (10.7, 13.3)	12.0 +2.0
Hematocrit (%)	36.7 (33.0, 40.3)	36.0 +6.3
Leukocyte Count (×10^3/μL)	10.0 (7.3, 12.9)	11.0 (6.6, 15.2)
Neutrophils (%)	84.0 (73.0, 89.0)	80.6 (71.0, 87.0)
Lymphocytes (%)	9.1 (6.0, 16.4)	11.1 (6.6, 19.0)
Platelet count (×10^3/μL)	120.0 (60.0, 194.5)	165.0 (114.0, 230.0)
Renal function		
Creatinine (mg/dL)	1.3 (0.9, 2.2)	1.0 (0.8, 1.3)
Blood urea nitrogen (mg/dL)	18.6 (13.0, 37.0)	14.0 (11.0, 22.0)
eGFR (mL/min/1.73 m^2)	68.7 (34.6, 94.0)	82.0 (56.9, 102.1)
Liver function		
Total bilirubin (mg/dL)	1.2 (0.7, 2.6)	1.0 (0.5, 2.3)
Direct bilirubin (mg/dL)	0.6 (0.3, 1.7)	0.5 (0.2, 1.5)
AST (U/L)	57.5 (35.0, 117.3)	63.0 (34.0, 152.5)
ALT (U/L)	51.0 (29.0, 86.8)	47.5 (29.0, 103.5)
Albumin (g/dL)	3.4 (2.8, 3.8)	3.6 (3.0, 4.0)
Metabolic parameters		
Bicarbonate (mmol/L)	23.0 (19.9, 25.5)	23.0 (21.0, 26.0)
Clinical outcomes		
ICU admission, n (%)	87 (19.3)	23 (13.7)
Mechanical ventilation, n (%)	54 (11.9)	10 (6.0)
Pulmonary hemorrhage, n (%)	48 (10.6)	7 (4.2)
Cardiovascular SOFA ≥3, n (%)	84 (18.6)	19 (11.3)
Coagulation SOFA ≥3, n (%)	113 (25.1)	19 (11.3)
Renal SOFA ≥3, n (%)	75 (16.7)	8 (4.8)
Hepatic SOFA ≥3, n (%)	43 (9.8)	13 (7.7)
In-hospital mortality, n (%)	25 (5.4)	5 (3.0)

Data are presented as mean ± standard deviation, median (interquartile range), or n (%), as appropriate. Laboratory-unconfirmed controls were defined as patients with clinically suspected leptospirosis who tested negative by all available laboratory-confirmatory methods.

Appendix 1 Table 3. ROC-Based Cutoff Values and Diagnostic Performance for Predicting In-Hospital Mortality

Factor	AUC (95% CI)	Cutoff value	Sensitivity (%)	Specificity (%)	PPV (%)	NPV (%)
Age	0.70 (0.60–0.80)	≥50 y	68.0	57.9	8.6	96.9
Total bilirubin	0.80 (0.70–0.90)	≥2 mg/dL	78.3	69.5	13.8	98.1
Leptospiremia	0.80 (0.70–0.90)	≥3.6 $\log_{10}$ copies/mL	76.0	73.9	14.5	98.1

Diagnostic performance metrics were obtained from ROC analysis, and cutoff values were determined using Youden's index. Sensitivity, specificity, positive predictive value (PPV), and negative predictive value (NPV) correspond to the ability of each marker to predict in-hospital mortality. AUC, area under the curve; CI, confidence interval; PPV, positive predictive value; NPV, negative predictive value.

Appendix 1 Table 4. Comparison of Baseline Characteristics and Clinical Outcomes Between the Overall Confirmed Cohort and the Genotyped Subset of Leptospirosis Patients

Variable	Overall confirmed cohort (n = 473)	Genotyped subset (n = 101)	p value
Demographics			
Age, years, median (IQR)	47.0 (35.0–59.0)	46.0 (33.0–59.3)	0.760
BMI (kg/m^2), median (IQR)	21.6 (19.5–24.2)	20.8 (18.4–23.8)	0.046*
Male sex, n (%)	389 (83.1)	79 (80.6)	0.551
Days from fever onset to admission, median (IQR)	3.0 (2.0–4.0)	3.0 (2.0–4.0)	0.555
Leptospiremia, log_{10} genome copies/mL (mean ± SD)	2.8 ± 1.4	4.1 ± 1.0	<0.001*
Clinical outcomes			
ICU admission, n (%)	87 (19.4)	37 (37.8)	<0.001*
Mechanical ventilation, n (%)	54 (11.9)	13 (13.1)	0.727
Pulmonary hemorrhage, n (%)	48 (10.6)	17 (17.2)	0.065
Cardiovascular SOFA ≥3, n (%)	84 (18.5)	39 (39.4)	<0.001*
Coagulation SOFA ≥3, n (%)	113 (24.9)	37 (37.8)	0.011*
Renal SOFA ≥3, n (%)	75 (16.6)	26 (26.5)	0.021*
Hepatic SOFA ≥3, n (%)	43 (9.7)	9 (9.5)	0.944
In-hospital mortality, n (%)	25 (5.5)	9 (8.9)	0.195

Data are presented as median (interquartile range, IQR), mean ± standard deviation (SD), or n (%).
Leptospiremia was quantified by real-time PCR targeting the lipL32 gene.
p values were calculated using the Mann–Whitney U test for continuous variables and χ^2 or Fisher exact test for categorical variables. *Indicates statistical significance at $p < 0.05$.

Appendix 1 Table 5. Characteristics of 13 *Leptospira interrogans* isolates included in whole-genome sequencing.

No	Code	Province	Fatal	AmpSeq	WGS Platform	CG272	Sequence Type
1	RLSS132	Sisaket		-	Illumina	Yes	ST 34 (7/7loci)
2	RLSS187	Sisaket		-	Illumina	No	ST 264 (7/7loci)
3	SSKH34	Sisaket		-	Illumina	Yes	ST 34 (6/7 loci)
4	LCBS02–204	Sisaket		Yes	PacBio	Yes	ST 34 (7/7loci)
5	LCBS02–141	Sisaket		Yes	Illumina	Yes	ST 34 (7/7loci)
6	LCBS02–120	Sisaket		Yes	Illumina	Yes	ST 34 (7/7loci)
7	LCBS02–201	Sisaket	Yes	Yes	Illumina	Yes	ST 34 (7/7loci)
8	LCBS02–219	Sisaket		Yes	Illumina	No	ST 76 (7/7loci)
9	LCBS02–111	Sisaket	Yes	Yes	Illumina	Yes	ST 34 (7/7loci)
10	LCBS02–108	Sisaket		Yes	Illumina	Yes	ST 34 (7/7loci)
11	RLSS039	Sisaket		-	Illumina	Yes	ST 34 (7/7loci)
12	RLSS046	Sisaket		-	Illumina	Yes	ST 34 (7/7loci)
13	RLSS056	Sisaket	Yes	-	Illumina	Yes	ST 34 (7/7loci)

Appendix 1 Table 6. Comparison of AmpSeq and WGS clonal group (CG) assignments among overlapping samples

No.	Sample ID	CG272 (AmpSeq)	CG272 (WGS)	Concordance	Comments
1	LCBS02/111	Yes	Yes	Yes	Consistent
2	LCBS02/120	Yes	Yes	Yes	Consistent
3	LCBS02/141	Yes	Yes	Yes	Consistent
4	LCBS02/201	Yes	Yes	Yes	Consistent
5	LCBS02/204	Yes	Yes	Yes	Consistent
6	LCBS02/219	No	No	Yes	Consistent
7	LCBS02/108	No	Yes	No	Possible mixed infection

AmpSeq and WGS data were compared for seven samples to assess concordance of CG classification. Six samples showed consistent CG272 assignment across both methods, while LCBS02–108 exhibited mixed reads in AmpSeq data, suggesting a possible mixed infection of L. interrogans strains. Abbreviations: AmpSeq, amplicon sequencing; WGS, whole-genome sequencing; CG, clonal group.

Appendix 1 Table 7. Comparison of CG272 and Non-CG272 *Leptospira interrogans* Infections

Variable	Overall (N = 84)	CG272 (n = 54)	Non-CG272 (n = 30)	P value
Demographics				
Age, years (mean ± SD)	47.8 ± 16.4	49.8 ± 17.2	43.9 ± 14.2	0.121
Male sex, n (%)	67 (81.7)	42 (77.8)	25 (89.3)	0.201
Alcoholism, n (%)	4 (4.9)	4 (7.4)	0 (0.0)	0.294
Cigarette smoking, n (%)	30 (37.0)	14 (25.9)	16 (59.3)	0.003
Vital signs and laboratory findings at admission				
Days from fever onset to admission, days (median, IQR)	3.0 (2.0, 4.0)	3.0 (2.0, 4.0)	3.0 (3.0, 4.0)	0.111
MAP, mmHg (mean ± SD)	73.5 ± 15.0	74.5 ± 15.2	71.7 ± 14.6	0.428
Platelet count, 10^3 cells/µL (median, IQR)	73.0 (32.8, 135.3)	69.5 (30.8, 133.8)	75.0 (45.0, 138.5)	0.852
Creatinine, mg/dL (median, IQR)	1.7 (1.2, 3.6)	1.7 (1.1, 2.9)	1.7 (1.2, 4.0)	0.575
Total bilirubin, mg/dL (median, IQR)	1.8 (0.8, 3.8)	1.7 (0.8, 3.7)	2.0 (0.8, 4.2)	0.843
Albumin, g/dL (mean ± SD)	3.2 ± 0.6	3.2 ± 0.7	3.1 ± 0.5	0.509
Bicarbonate, mEq/L (median, IQR)	21.0 (18.0, 23.0)	21.0 (17.5, 23.5)	19.4 (18.0, 23.0)	0.777
Leptospiremia, log_{10} genome copies/mL (mean ± SD)	4.2 ± 1.1	4.3 ± 1.1	4.0 ± 1.1	0.198
Clinical outcomes				
ICU admission, n (%)	36 (44.4)	24 (46.2)	12 (41.4)	0.678
Mechanical ventilation, n (%)	13 (15.9)	7 (13.2)	6 (20.7)	0.528
Pulmonary hemorrhage, n (%)	16 (19.5)	10 (18.9)	6 (20.7)	0.842
Cardiovascular SOFA ≥3, n (%)	38 (46.3)	23 (43.4)	15 (51.7)	0.470
Coagulation SOFA ≥3, n (%)	36 (44.4)	26 (50.0)	10 (34.5)	0.178
Renal SOFA ≥3, n (%)	24 (29.6)	14 (26.9)	10 (34.5)	0.475
Hepatic SOFA ≥3, n (%)	9 (11.4)	5 (9.8)	4 (14.3)	0.713
Multiorgan dysfunction, n (%)	39 (46.4)	24 (44.4)	15 (50.0)	0.625
Number of organ involvement (median, IQR)	1.0 (0.0,3.0)	1.0 (0.0,3.0)	1.5 (0.0,3.0)	0.733
In-hospital mortality, n (%)	9 (10.7)	9 (16.7)	0 (0.0)	0.023
Length of stay, days (median, IQR)	5.0 (3.0, 9.0)	4.5 (3.0, 9.0)	6.0 (3.0, 9.0)	0.438

Data are presented as mean ± standard deviation (SD), median with interquartile range (IQR), or number (percentage). P values represent comparisons between CG272 and non-CG272 infections using the chi-square or Fisher exact test for categorical variables and the Mann–Whitney U test or t-test for continuous variables, as appropriate. MAP, mean arterial pressure; SOFA, Sequential Organ Failure Assessment; ICU, intensive care unit; IQR, interquartile range; SD, standard deviation.

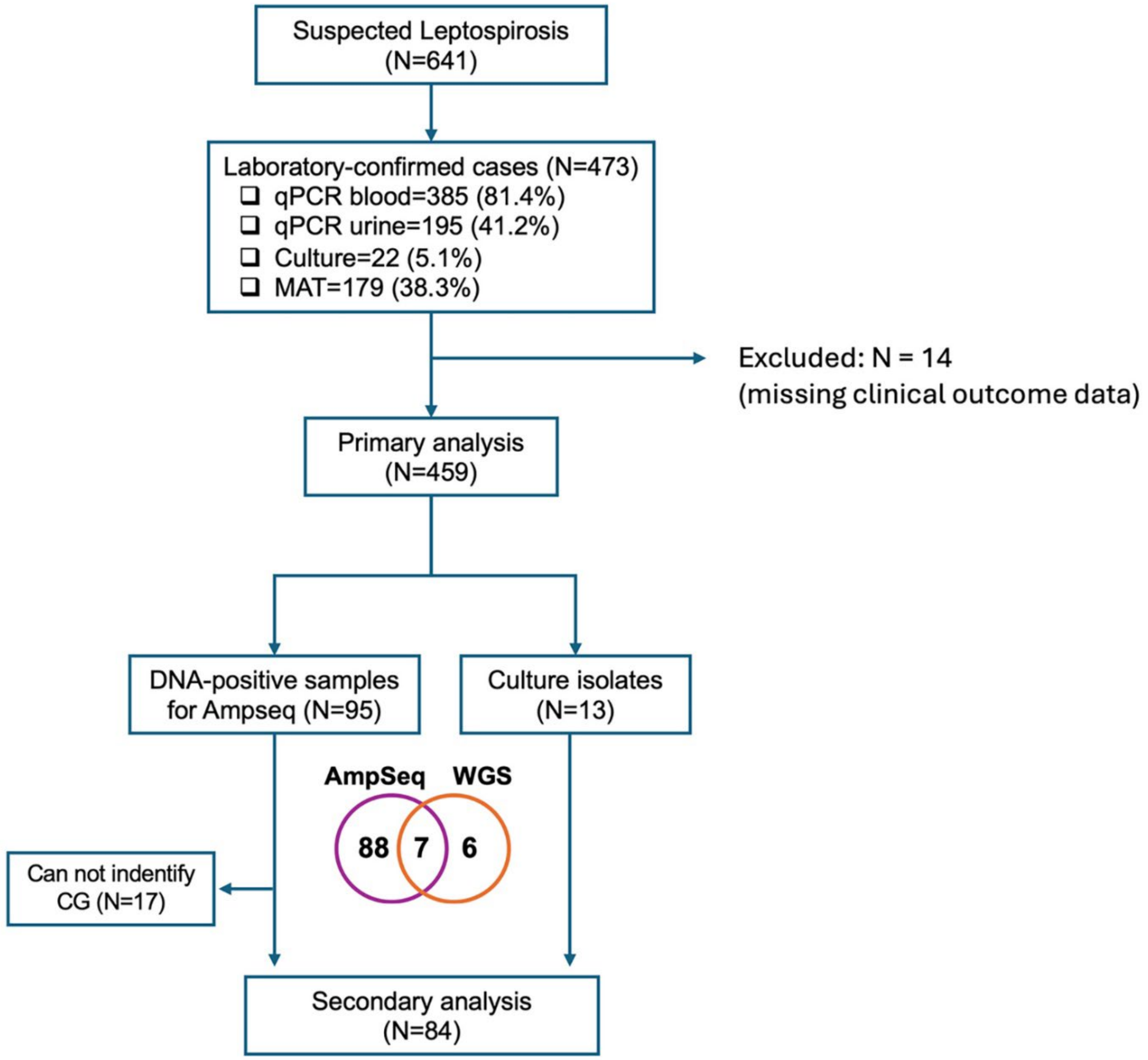

Appendix 1 Figure 1. Study workflow and sample selection for primary and secondary analyses. The diagram outlines the overall study population and the derivation of samples included in the primary and genomic secondary analyses. A total of 641 patients with suspected leptospirosis were screened. Of 473 laboratory-confirmed cases, 14 were excluded due to missing clinical outcome data, leaving 459 cases for the primary clinical analysis. For the secondary genomic analysis, 95 qPCR-positive DNA samples underwent AmpSeq, and 13 culture isolates underwent whole-genome sequencing (WGS). Seven samples were analyzed by both methods, and 84 samples had sufficient data to allow clonal-group (CG) classification (e.g., CG272 versus non-CG272). Seventeen AmpSeq samples were sequenced but could not be assigned to a clonal group due to insufficient coverage.

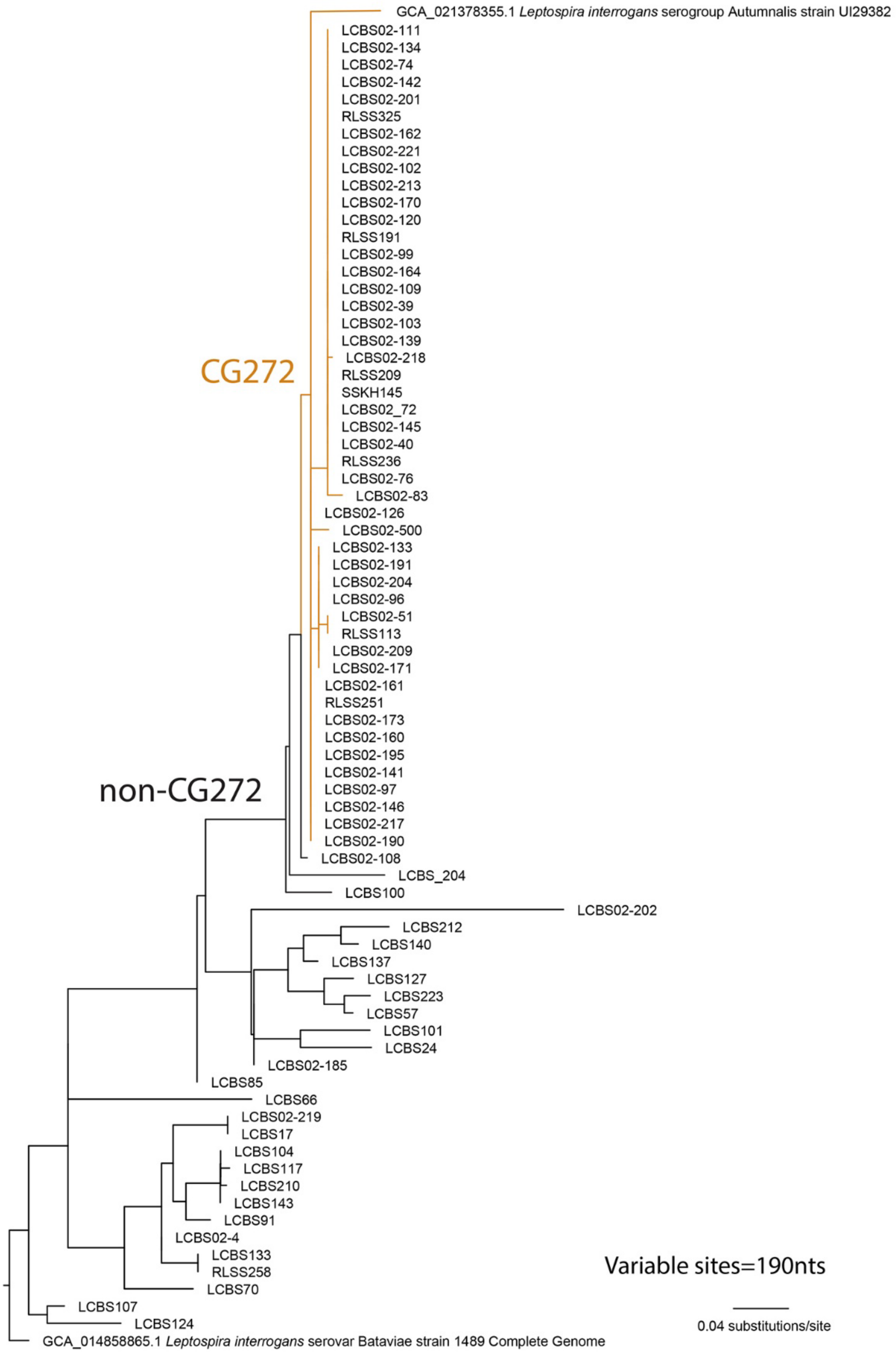

Appendix 1 Figure 2. Phylogenetic analysis of *Leptospira interrogans* from AmpSeq data. A maximum-likelihood phylogenetic tree was constructed from 190 variable sites among 76 AmpSeq-analyzed *L. interrogans* sequences. Samples segregated into two major lineages corresponding to CG272 and non-

CG272 clades. CG272 formed a tightly clustered clade with strong phylogenetic support, whereas non-CG272 samples were more genetically diverse. CG272 and non-CG272 lineages are color-coded in gold and black. All fatal cases included in the AmpSeq dataset were located within the CG272 clade. Scale bar represents 0.04 nt substitutions per site.
